# Soluble ICAM-5, a Product of Activity Dependent Proteolysis, Increases mEPSC Frequency and Dendritic Expression of GluA1

**DOI:** 10.1371/journal.pone.0069136

**Published:** 2013-07-02

**Authors:** Irina Lonskaya, John Partridge, Rupa R. Lalchandani, Andrew Chung, Taehee Lee, Stefano Vicini, Hyang-Sook Hoe, Seung T. Lim, Katherine Conant

**Affiliations:** 1 Department of Neuroscience, Georgetown University Medical Center, Washington, D.C., United States of America; 2 Department of Pharmacology and Physiology, Georgetown University Medical Center, Washington, D.C., United States of America; 3 Interdisciplinary Program in Neuroscience, Georgetown University Medical Center, Washington, D.C., United States of America; University of Nebraska Medical Center, United States of America

## Abstract

Matrix metalloproteinases (MMPs) are zinc dependent endopeptidases that can be released from neurons in an activity dependent manner to play a role in varied forms of learning and memory. MMP inhibitors impair hippocampal long term potentiation (LTP), spatial memory, and behavioral correlates of drug addiction. Since MMPs are thought to influence LTP through a β_1_ integrin dependent mechanism, it has been suggested that these enzymes cleave specific substrates to generate integrin binding ligands. In previously published work, we have shown that neuronal activity stimulates rapid MMP dependent shedding of intercellular adhesion molecule-5 (ICAM-5), a synaptic adhesion molecule expressed on dendrites of the telencephalon. We have also shown that the ICAM-5 ectodomain can interact with β_1_ integrins to stimulate integrin dependent phosphorylation of cofilin, an event that occurs with dendritic spine maturation and LTP. In the current study, we investigate the potential for the ICAM-5 ectodomain to stimulate changes in α-amino-3-hydroxyl-5-methyl-4-isoxazole-propionate receptor (AMPAR) dependent glutamatergic transmission. Single cell recordings show that the ICAM-5 ectodomain stimulates an increase in the frequency, but not the amplitude, of AMPA mini excitatory post synaptic currents (mEPSCs). With biotinylation and precipitation assays, we also show that the ICAM-5 ectodomain stimulates an increase in membrane levels of GluA1, but not GluA2, AMPAR subunits. In addition, we observe an ICAM-5 associated increase in GluA1 phosphorylation at serine 845. Concomitantly, ICAM-5 affects an increase in GluA1 surface staining along dendrites without affecting an increase in dendritic spine number. Together these data are consistent with the possibility that soluble ICAM-5 increases glutamatergic transmission and that post-synaptic changes, including increased phosphorylation and dendritic insertion of GluA1, could contribute. We suggest that future studies are warranted to determine whether ICAM-5 is one of a select group of synaptic CAMs whose shedding contributes to MMP dependent effects on learning and memory.

## Introduction

Matrix metalloproteinases (MMPs) are a family of structurally related enzymes that can be released from cells as pro- and active forms. They were named for their ability to process proteins of the extracellular matrix but are now appreciated to act on a variety of soluble molecules and cell surface receptors as well [1]. While studies of MMPs in the CNS have generally focused on the potential for pathologically elevated enzyme levels to stimulate blood brain barrier breakdown or cellular injury, recent evidence suggests that physiological levels of select MMPs can play a critical role in normal CNS function and learning and memory in particular [2–4]. For example, several groups have shown that MMPs are important to spatial learning and memory, and to correlates of the maladaptive memory that underlies addiction [5,6]. Previous studies have also shown that MMP inhibitors can impair LTP [7,8].

Consistent with a role for MMPs in learning and memory, expression and release of the enzymes can be increased by neuronal activity [9–12]. Such release may be rapid, in that MMP dependent shedding of a neuronal substrate occurs within several minutes of N-methyl-D-aspartic acid (NMDA) application [11]. Published studies suggest that preformed MMPs exist in perisynaptic stores [12,13], and in non neural cells, stimulated release can follow from a soluble NSF attachment protein receptor (SNARE) dependent mechanism [14]. If a similar mechanism occurs in neurons, MMP release might be facilitated by stimuli that evoke SNARE dependent release of select neurotransmitters. A recent study has also shown that glutamate stimulates transport of MMP-9 mRNA to dendrites, and that neuronal activity stimulates local translation and release of the enzyme [15].

The ability of MMPs to influence long term potentiation and hippocampal dependent memory likely involves structural changes to the post synaptic element of glutamatergic synapses [16]. More than 90% of excitatory synapses terminate on dendritic spines [17], and long lasting facilitation of neurotransmission has been linked to increases in the size of spines and associated increases in the number of glutamate receptors [18–20]. Consistent with the potential for MMPs to influence dendritic spines, at least one MMP has been shown to increase spine size [21]. The means by which MMPs exert their effects on dendritic spines and LTP are, however, not completely understood. Previous studies suggest that the engagement of β_1_ integrins may contribute [8]. Integrins including β_1_ are expressed at the synapse, integrin activation plays a role in LTP, and integrin antagonists can block MMP-dependent LTP and spine enlargement [8,21–27]. Engagement of β_1_ integrin receptors has been shown to stimulate src kinase dependent phosphorylation of NMDA receptors [23], and may also stimulate the actin polymerization that underlies spine expansion [22].

In terms of how MMP activity stimulates integrin dependent effects, one possibility is that MMPs cleave specific synaptic cell adhesion molecules (CAMs) to generate integrin binding ligands. Varied CAMs are known to possess integrin binding domains [28], and several of these are CAMs are enriched at the glutamatergic synapse [29]. CAMs are also well localized to be MMP substrates, in that their proximity to sites of MMP release may allow them to be cleaved before MMPs are bound by endogenously expressed MMP inhibitors, or tissue inhibitors of metalloproteinases (TIMPs).

In a previously published study, we have shown that neuronal activity stimulates rapid MMP-dependent cleavage of the synaptic cell adhesion molecule intercellular adhesion molecule-5 (ICAM-5), an adhesion molecule that is highly expressed on dendrites of the telencephalon [11,30]. Earlier studies had shown that ICAM-5 shedding was associated with spine maturation [29,30]. These studies, which focused on developmental spine maturation and evaluated spine morphology many hours following NMDA-stimulated ICAM-5 cleavage [29], had suggested that the shedding of ICAM-5 might disrupt N and C terminal interactions of the full length molecule that are important to filopodial maintenance [31]. While shedding may therefore allow for spine expansion, a non-mutually exclusive possibility, and one that we have focused on in recent studies [32], is that the shed ectodomain can bind to unengaged post synaptic integrins to stimulate dendritic actin polymerization and spine expansion.

In a previous publication [33], we have shown that the ectodomain of ICAM-5 can interact with β_1_ integrins to stimulate phosphorylation of cofilin, an event associated with dendritic actin polymerization. The question of whether soluble ICAM-5 dependent effects are significant enough to influence dendritic levels of glutamate receptors and AMPA mEPSCs is addressed in the current study.

## Materials and Methods

### Cell Culture

All experimental procedures were approved by and performed in agreement with policies of the Georgetown University Animal Care and Use Committee (GUACUC). Every effort was made to minimize suffering. Hippocampal tissue was harvested from embryonic day 18 Sprague-Dawley rats using a protocol modified from [34]. Briefly, hippocampal tissue was finely chopped and digested with 0.1% trypsin as well as by mechanical trituration. Cells were plated onto cell culture-ware previously treated with poly-d-lysine and laminin (Sigma, St. Louis, MO), at an approximate density of 150 cells/mm^2^. Cultures were maintained in Neurobasal A medium with B27 (Invitrogen, Carlsbad, CA), with bi-weekly changes, and stored in a humidified 5% CO_2_ and 95% O_2_ incubator at 37°C. Experiments were performed on cultures at 14 days *in vitro* (DIV).

### Reagents

Recombinant ICAM-5 was purchased from R & D Systems, Minneapolis, MN and reconstituted in sterile phosphate buffered saline just prior to use. This construct contains the major portion of the ICAM-5 ectodomain (leu 31-arg828). Antibodies to GluA1 were purchased from Millipore (AB1504, C terminal epitope) and Calbiochem (PC246, N terminal epitope for live surface staining). The anti-phospho-GluA1 was from from R & D Systems (PPS008), the anti-GluA2 from BD pharmingen (live surface staining) and Millipore/Chemicon (Western blot following biotinylation/precipitation), and the anti-PSD95 from Millipore/Chemicon (MAB1598). The pEGFP construct was commercially obtained (Clonetech), and Lipofectamine 2000 was purchased from Invitrogen.

### Cell stimulation

ICAM-5 treated cultures received 1-2.5 µg/ml (as noted) recombinant protein for 60 min prior to analysis. It was previously established that this concentrations in this range stimulated an increase in phospho-cofilin [33].

### Single Cell Recordings

Ruptured-patch whole-cell voltage-clamp recordings were obtained from cultured hippocampal neurons at DIV 12-14 for examination of glutamate receptor responses. Pyramidal cells were selected using a 60X water immersion objective with a long working distance (2 mm) and high numerical aperture (1.0). Recording electrodes (4-6 MΩ tip resistance) were pulled on a vertical pipette puller from borosilicate glass capillaries (Wiretrol II, Drummond, Broomall, PA) and filled with an internal solution containing (in mM): 145 K-gluconate, 10 HEPES, 5 ATP-Mg, 0.2 GTP-Na, and 0.5 EGTA, adjusted to pH 7.2 with KOH. Extracellular solution was perfused at a rate of 2.0-2.8 ml/minute and contained (in mM): 145 NaCl, 5 KCl, 1 CaCl_2_, 5 HEPES, 5 glucose, 26 sucrose and 0.25 mg/L phenol red, adjusted to pH to 7.4 with NaOH. Voltage-clamp recordings were performed at a holding potential of -70 mV using either a Multiclamp 700B or an Axopatch 1-D amplifier (Molecular Device Co., Sunnyvale CA, USA).

Miniature excitatory postsynaptic currents (mEPSCs) were isolated by local application of 25 μM of bicuculline methobromide (BMR) and 0.5 μM tetrodotoxin (TTX) through the “Y tube” method [35]. The AMPA receptor antagonist NBQX (1,2,3,4-tetrahydro-6-nitro-2,3-dioxo-benzo[f]quinoxaline-7-sulfonamide disodium salt hydrate) was applied in a subset of recordings to verify events were AMPA receptor mediated. All drug-containing stock solutions were diluted to the desired working concentration in the extracellular solution.

Currents were low pass filtered at 2 kHz and digitized at 5-10 kHz using a Dell computer equipped with Digidata 1322A data acquisition board and pCLAMP9 software (Molecular Devices). Events were identified using a semi-automated threshold based mini detection software (Mini Analysis, Synaptosoft Inc., Fort Lee, NJ) and were visually confirmed. mEPSC averages were based on >100 events in each recording.

### Sample preparation and Western blot

Western blot was performed on lysates as previously described [33]. Lysates from cultured cells were prepared via the addition of lysis buffer [50 mM Tris–HCl, pH 7.5, 150 mM NaCl, 0.1% sodium dodecyl sulfate, 1% NP-40, 0.5% sodium deoxycholate, 0.2 mM phenylmethylsulfonyl fluoride, 0.5 mM dithiothreitol, 1× protease inhibitor cocktail (Sigma P8340)]. The mixture was placed into a microfuge tube, sonicated for 10 s, kept on ice for 20 min, and then spun at 14 000 rpm for 15 min at 4°C in a microcentrifuge. The quality of transfer was verified by Ponceau staining and molecular weights were inferred by comparison to prestained markers (BioRad).

For analysis of surface GluA1 and GluA2, surface proteins were first biotinylated, and then pulled down to be analyzed by Western blot. Cultures were treated for 1 hour after which they were washed twice with cold phosphate-buffered saline and incubated in PBS containing 1 mg/ml EZ-Link Sulfo-NHS-Biotin (Pierce) for 30 min at 4 °C. The biotinylation reaction was stopped by washing cells with quenching solution (PBS/100 mM glycine). Cells were incubated in quenching solution for a total of 20 min. Cells were then solubilized at 4 °C in a lysis buffer, containing 150 mM NaCl, 1 mM EDTA, and 100 mM Tris-HCl, pH 7.4 1% Triton X-100, and protease inhibitor cocktail (Roche). To clear lysates, samples were spun at 16 000 × *g* at 4 °C for 20 min. A small portion of cleared lysate was saved for analysis, as a lysate fraction. The remaining lysate was incubated with avidin beads (Pierce) at 4 °C overnight. After incubation, beads were pelleted by centrifugation at 16 000 × *g* for 15 min, and the supernatant was saved as the intracellular fraction. The beads were washed once in lysis buffer, and then twice in lysis buffer containing high salt (500 mM NaCl), and once again in lysis buffer containing low salt (50 mM NaCl). Biotinylated proteins were eluted with SDS sample buffer, containing 100 mM mercaptoethanol. The integrity of the cell membrane during biotinylation was tested by immunoblotting with an anti-actin antibody.

### Immunocytochemistry, Microscopy, and Image Analysis

Live cell surface staining was performed as described [36–38]. Briefly, neurons were transfected with pEGFP 24 h before initiation of experiments. At the completion of each experiment, neurons were incubated with primary antibody for 10 minutes then lightly fixed for 5 minutes in 4% paraformaldehyde (nonpermeabilizing conditions). After fixation, antibody labeled GluA1 or GluA2 was detected with Alexa Fluor linked secondary antibody.

For quantitative studies of surface staining, spine and filopodia analysis and puncta numbers, secondary dendrites from 16–32 pyramidal neurons were evaluated per experimental group. Each group included three replicate cover slips. 20 µm segments beginning 10 microns from the soma were evaluated. Quantitation of surface signal and puncta were performed as previously described [36,37]. Spines and filopodia were defined as structures having a length of 0.2-2 µm and greater than 2 µm respectively.

## Results

### I. Soluble ICAM-5 stimulates an increase in mEPSC frequency

**Figure 1:**
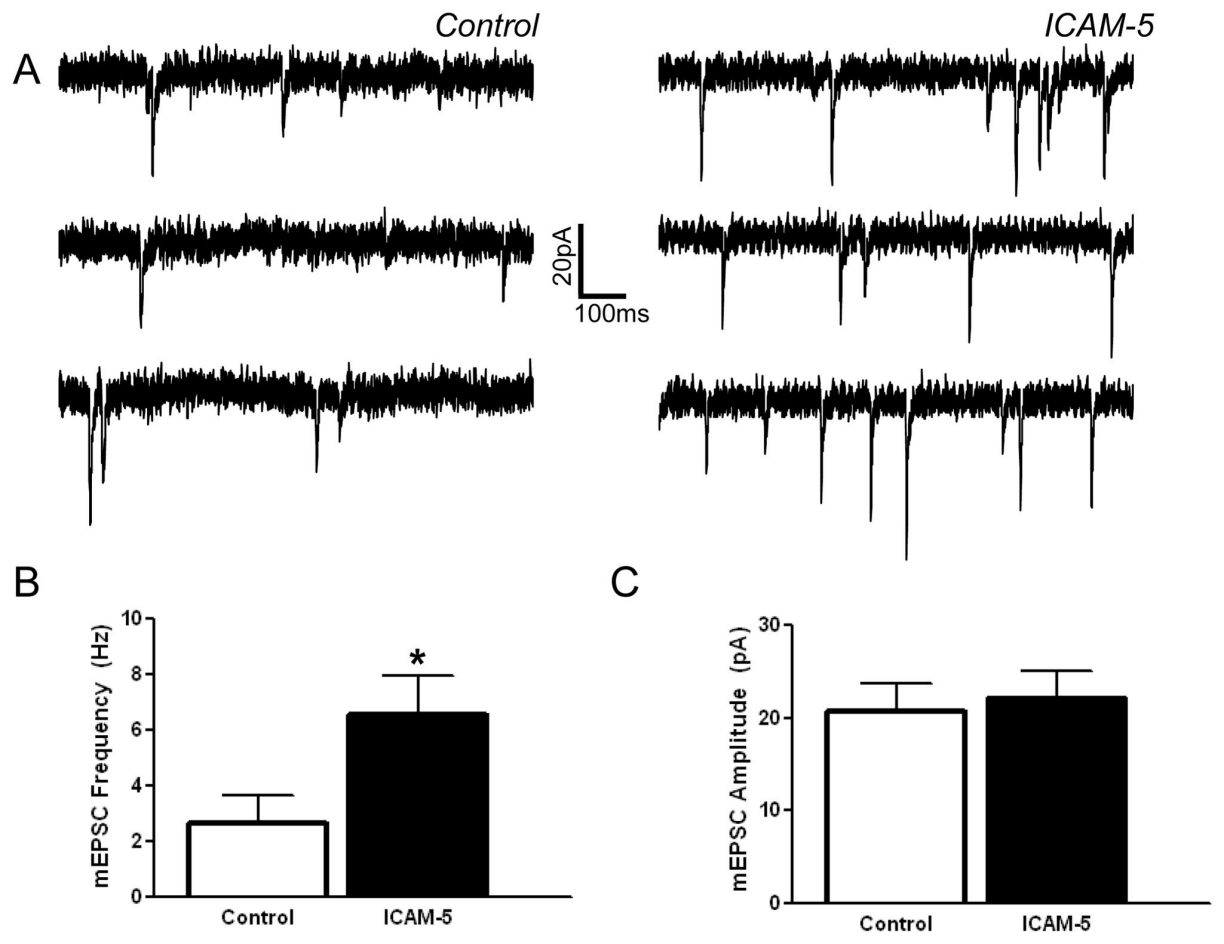
The ICAM-5 ectodomain affects an increase in mini excitatory post synaptic current (mEPSC) frequency. Stimulation of rat hippocampal neurons with 1 µg/ml ICAM-5 ectodomain (60 min. pretreatment) is associated with an increase in mEPSC frequency. In these experiments, 1,468 events from 11 control cells and 2353 events from 16 ICAM-5 stimulated cells were evaluated using standard techniques [74]. Representative tracings are shown in (A) while the average mEPSC frequency is shown in (B) and amplitude in (C). The difference between mEPSC frequency in control and ICAM treated neurons was significant (*p < 0.05, Student’s *t* test).

ICAM-5 is expressed on dendrites of the telencephalon and shed in a neuronal activity dependent manner. Its shedding by MMPs generates an N terminal fragment containing the major portion of the ectodomain including integrin binding domains [11,26]. In previous studies, we have shown that soluble ICAM-5 co-immunoprecipitates with β_1_ integrins [33]. We have also shown that it can stimulate a β_1_ integrin dependent increase in action potential frequency, an endpoint that can be associated with changes including, but not limited to, altered frequency or amplitude of AMPAR mEPSCs [32]. Herein we have evaluated the potential for soluble ICAM-5 to influence AMPAR mEPSC recordings in hippocampal neurons. Representative tracings are shown in Figure 1A. As shown in Figure 1B, we see an ICAM-5 stimulated increase in the frequency of AMPAR mEPSCs. Of interest, an increase in the amplitude of mEPSCs is not observed (Figure 1C).

### II. Soluble ICAM-5 stimulates an increase in surface levels of GluA1

**Figure 2:**
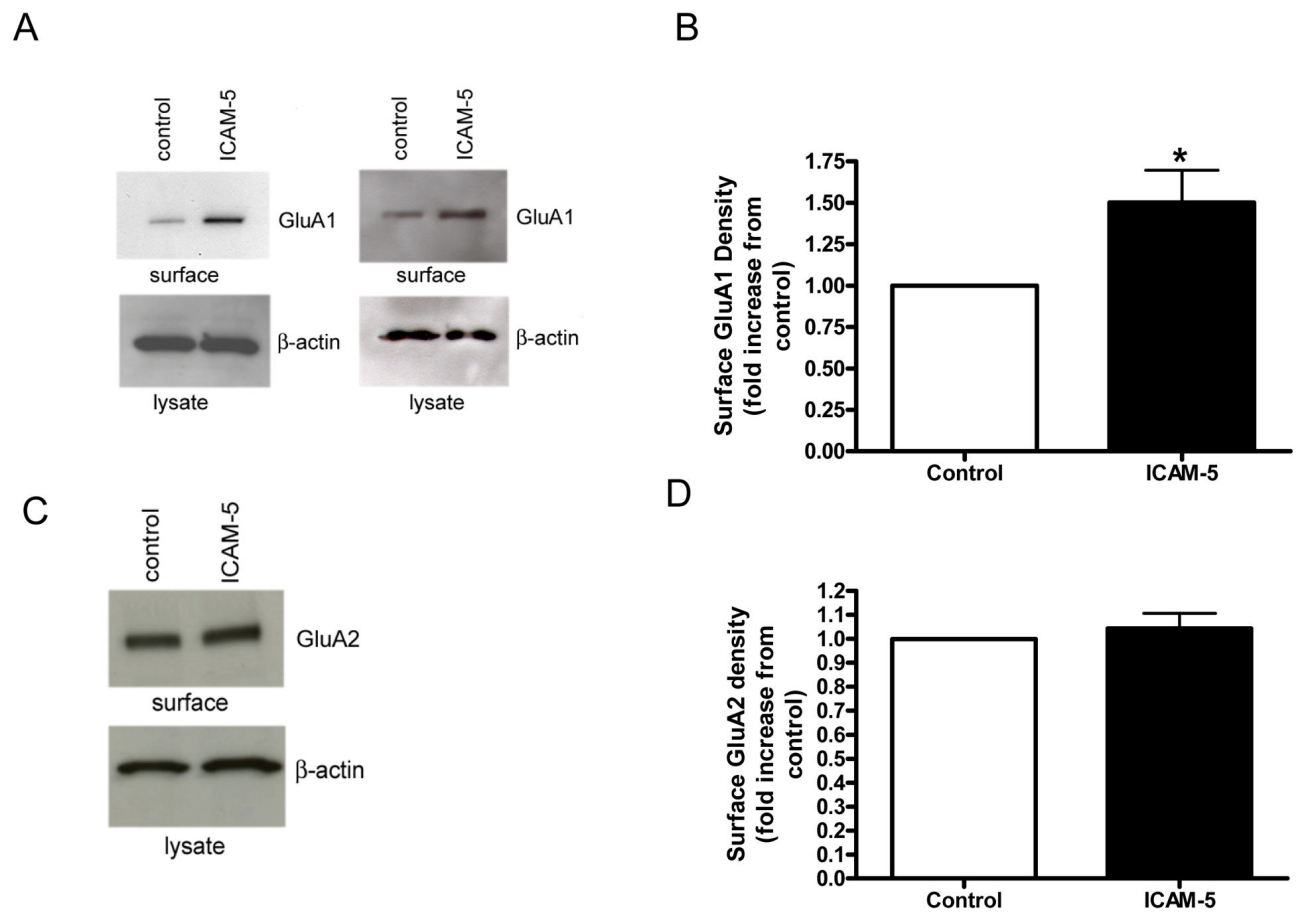
The ICAM-5 ectodomain stimulates an increase in surface levels of the glutamate receptor subunit GluA1. Rat hippocampal neurons were unstimulated (control) or stimulated for 60 min. with 1 µg/ml of the ICAM-5 ectodomain (R & D Systems). Surface proteins were then biotinylated, and biotinylated proteins pulled down to be analyzed by Western blot. As can be appreciated, ICAM-5 was associated with an increase in surface GluA1 (A). Blots from separate experiments are shown. Densitometric analysis showing the fold increase in GluA1 band intensity in ICAM-5 versus control treated cultures in shown in (B). The mean and standard error for the fold increase from 6 replicate experiments is shown, and the difference between control and ICAM-5 groups is significant at *p* < 0.1 (*p=0.05). A representative blot for GluA2 in surface protein preparations is shown in (C), and densitometric analysis showing the fold change in GluA2 band intensity from 3 replicate experiments follows in (D). The mean and standard error for the fold change from 3 replicate experiments is shown, and the difference between control and ICAM-5 groups is not significant (*p*= 0.6).

Several studies suggest that MMPs can potently and rapidly modulate the structure of dendritic spines in particular, with overall effects likely influenced by the particular MMP family member, its concentration, and the maturity of the system studied [21,39,40]. A potential post synaptic contribution to the change in mEPSC frequency could follow from an increase in the number of AMPA responsive synapses. We therefore tested ICAM-5 for its ability to influence surface levels of GluA1 and 2. As shown in Figure 2A, ICAM-5 was associated with an increase in surface levels of the GluA1 receptor subunit. Figure 2B shows results of densitometric analysis from replicate experiments. While there was variability in the increase, there was an ICAM-5 associated increase in each experiment. Actin levels in lysates did not differ, nor did total lysate levels of GluA1 (not shown). In figure 2C, surface protein preparations that had shown ICAM-5 associated changes in GluA1 were also examined for GluA2. While this subunit is also important to LTP [20], at one hour post treatment we did not observe an associated increase in surface levels of GluA2.

### III. Soluble ICAM-5 is associated with an increase in the phosphorylation of GluA1 at serine 845

**Figure 3:**
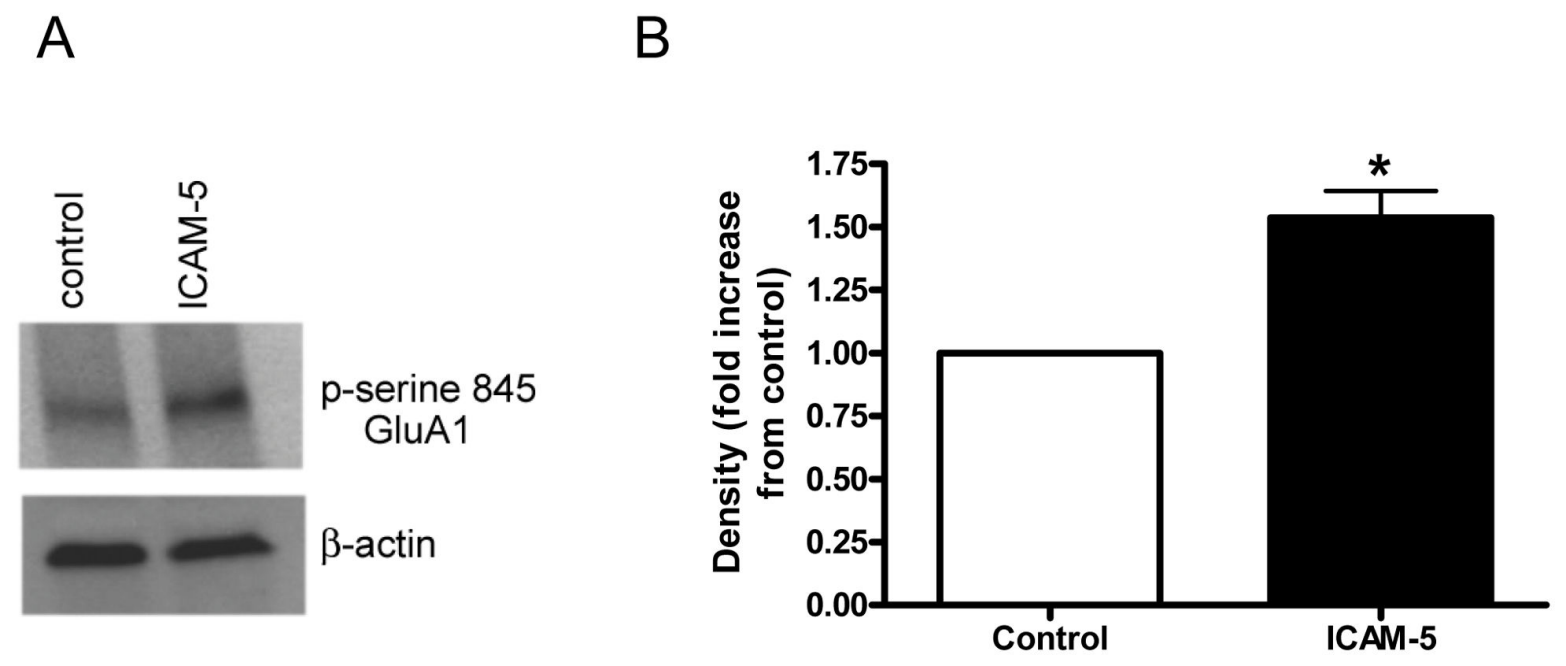
Phosphorylation of GluA1 at serine-845 is increased by soluble ICAM-5. Rat hippocampal neurons were unstimulated (control) or treated for 60 min. with 1 µg/ml of soluble ICAM-5 and lysates tested by Western blot for phospho-serine 845 GluA1. A representative blot is shown in (A) while densitometric analysis of blots from 5 experiments using distinct cultures is shown in (B). The mean and standard error for the fold increase is shown, and the fold increase is significant at *p* < 0.1 (*p= 0.06).

Phosphorylation of GluA subunits can influence receptor function and subunit localization 845 [41–43]. GluA1 phosphorylation sites include serine 897 and serine 845, with the latter typically stimulated by PKA. PKA dependent phosphorylation has been linked to activity dependent synaptic incorporation of the subunit [42], and the serine 845 site linked to fear memory [44]. Importantly, β_1_ integrin agonists have recently been shown to associate with Gαs and activate cAMP/PKA [45]. In figure 3, we show results from experiments that examined the ability of soluble ICAM-5 to stimulate an increase in the serine 845 phosphorylation of GluA1. Results from representative Western blot are shown in Figure 3A, and results from densitometric analysis of blots from 5 experiments are shown in Figure 3B.

### IV. ICAM-5 affects an increase in GluA1 surface staining along dendrites

**Figure 4:**
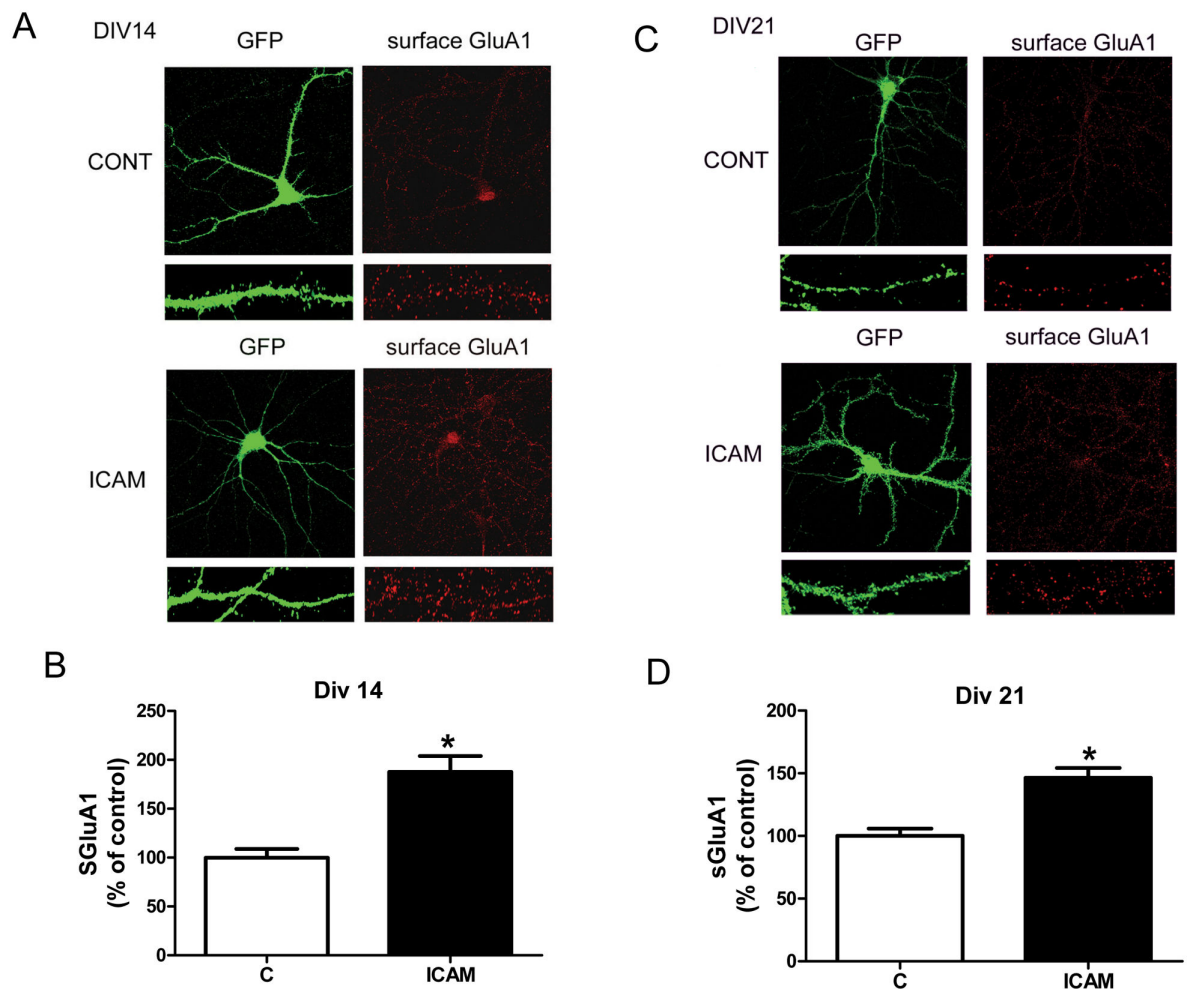
Soluble ICAM-5 affects an increase in GluA1 surface staining along dendrites in particular. Figure 4 shows data from live cell surface staining for GluA1 in control and ICAM-5 treated hippocampal neurons at 14 and 21 DIV. Indicated cultures were treated with 2.5 µg/ml soluble ICAM-5 and surface staining performed 1 hour later. Representative images are shown in A and C, while quantitative data is shown in B and D. The mean and standard error for percent control values were 100 +/- 8.8, n=21 for the DIV 14 control group; 187.5 +/- 16.4, n=16 for the DIV 14 ICAM-5 group; 100 +/- 5.8, n=25 for the DIV 21 control group; and 146.3 +/- 7.9, n=25 for the DIV 21 ICAM-5 group. Differences in GluA1 staining between control and ICAM-5 treated cultures are significant at *p*< 0.01 (*) at both 14 and 21 DIV.

An increase in surface GluA1 as detected by biotinylation and precipitation assays is not localization specific. To determine whether surface GluA1 increased along dendrites in particular, surface labeling studies were performed according to established methods [37]. Results are shown in Figure 4 and demonstrate an increase in the intensity of GluA1 along proximal dendritic spines in ICAM-5 treated cultures at both 14 and 21 DIV. The intensity of GluA2 was not increased by ICAM-5, showing instead a non significant decrease at DIV 14 (not shown).

### V. Soluble ICAM-5 does not affect an increase in dendritic spine number

**Figure 5:**
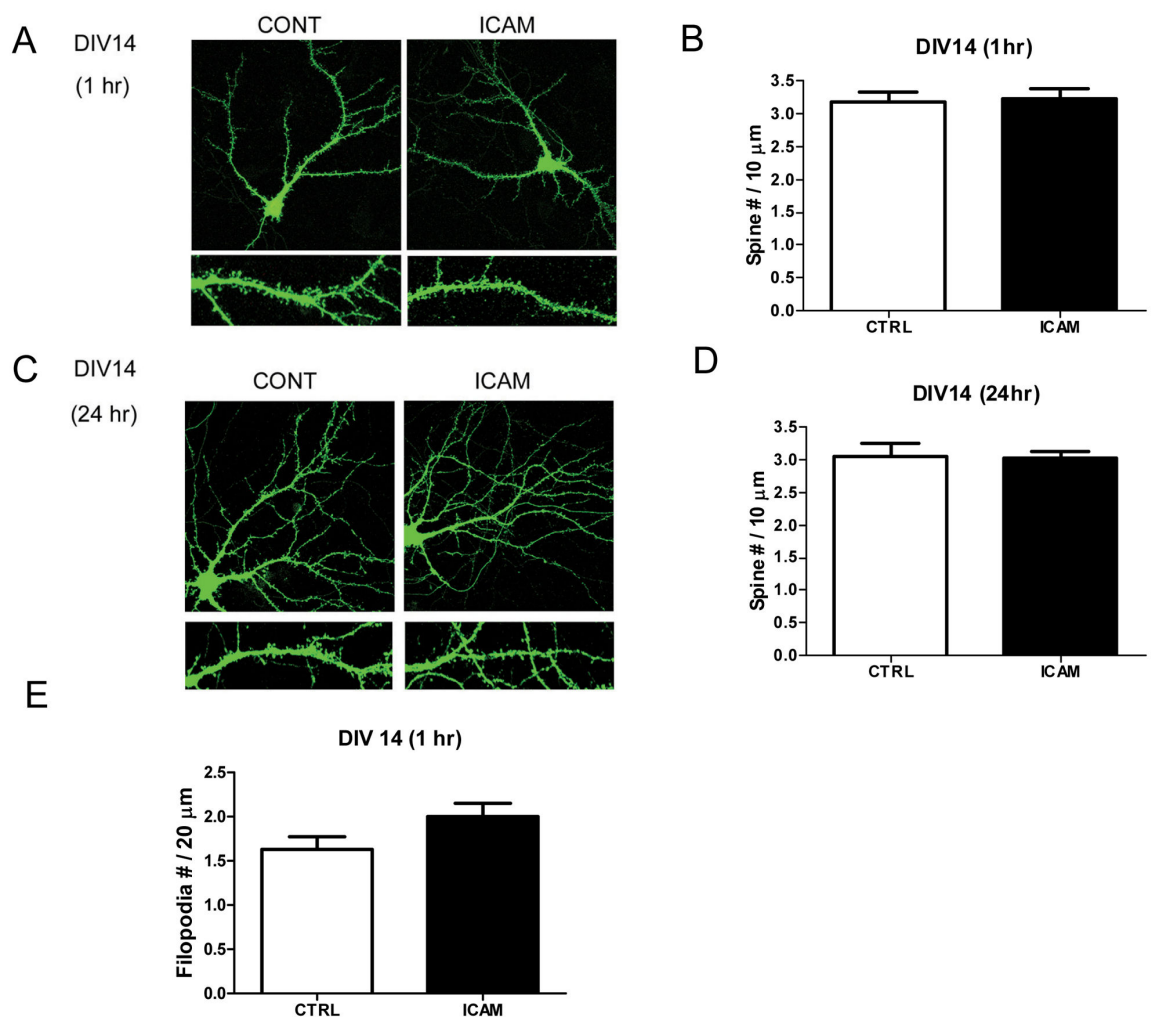
Soluble ICAM-5 does not increase spine number. Figure 5 shows results from and experiment that compared dendritic spine number in control and ICAM-5 treated cultures. In cultures that were treated for 1h or 24h with 2.5 µg/ml ICAM-5, there was no significant difference in spine number (A–D). There was, however, a non-statistically significant trend towards an increase in dendritic filopodia, defined as protrusions greater than 2 µm in length, in cultures treated for 1h with ICAM-5 (E). The mean and standard error for spine number was 3.172 +/- 0.15, n=30 for the 1h control group; 3.23 +/- 0.149, n=32 for the 1h ICAM-5 group; 3.065 +/- 0.2, n=24 for the 24 h control group; and 3.027 +/-0.11, n=31 for the 24h ICAM-5 group. The mean and standard error for filopodia number were 1.6 +/- 0.14, n=27 for the control group and 2 +/- 0.15, n=27 for the ICAM-5 group.

An increase in mEPSC frequency and dendritic surface levels of GluA1 could follow, at least in some part, from an increase in the insertion of GluA1 into existing, but GluA lacking and thus post synaptically silent, synapses. While increased staining for GluA1 along dendrites could follow from increased insertion along the shaft and/or increased insertion into existing synapses, a non-mutually exclusive possibility that could contribute to increased staining and an increase in the number of AMPAR responsive synapses would be an increase in spine number with GluA1 entering newly formed spines. Of interest is that in certain brain regions, *de novo* spines can form quickly [46]. Therefore, we also tested sICAM-5 for its effects on dendritic spine number. As shown in Figure 5, however, ICAM-5 did not stimulate a significant increase in spine number. Of interest, is that a non-significant trend towards an increase in filopodia was observed.

### VI. Schematic representation of MMP-dependent ICAM-5 signaling at the synapse

**Figure 6:**
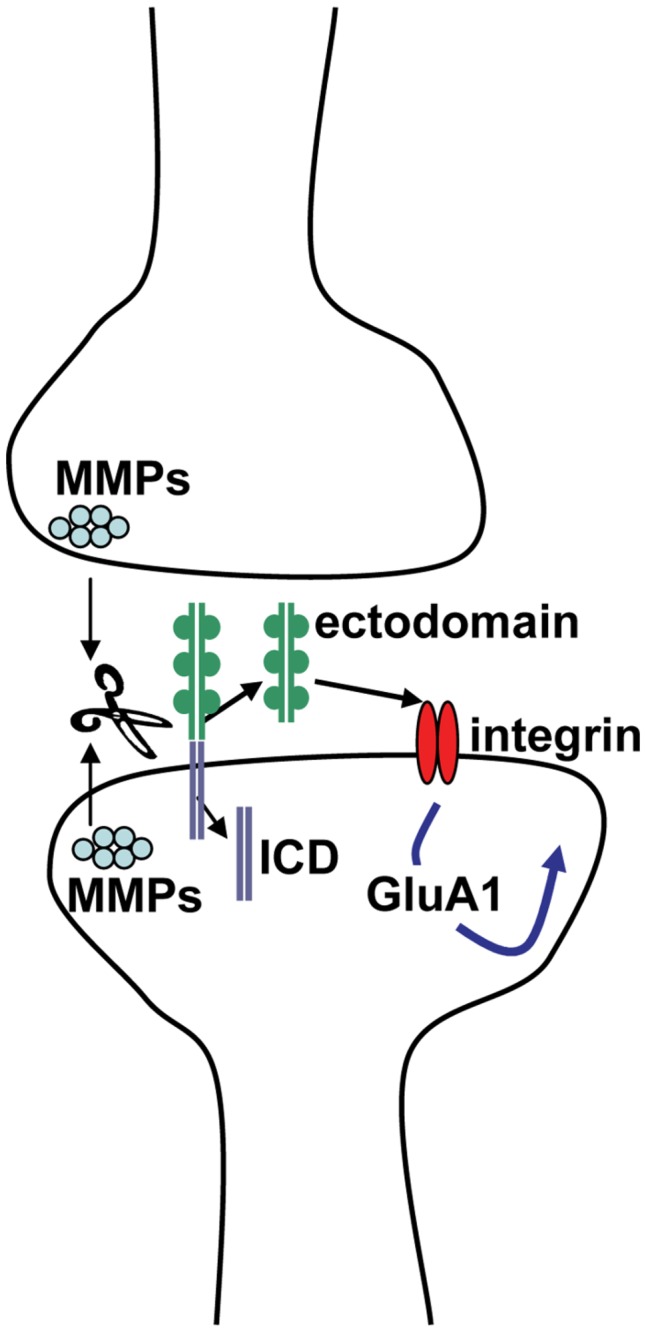
Schematic representation of MMP-dependent ICAM-5 signaling at the synapse. In figure 6 we show a hypothetical model in which MMPs are rapidly released from preformed peri-synaptic stores to cleave ICAM-5 (green and lavender) at a membrane proximal site. The released N terminal fragment can bind unengaged integrins (red ovals) to stimulate intracellular signaling cascades leading to increased phosphorylation and membrane insertion of GluA1 subunits. Following ectodomain shedding, the C terminal fragment of ICAM-5 could undergo additional processing followed by internalization and degradation. It is worth noting that following MMP or ADAM mediated shedding, select CAMs are further processed by intramembranous proteolysis. ICDs thus generated may be degraded or, in some cases, influence gene transcription.

In figure 6 we show a hypothetical model in which MMPs are rapidly released from preformed peri-synaptic stores to cleave ICAM-5 at a membrane proximal site. The released N terminal fragment can bind unengaged integrins to stimulate intracellular signaling cascades leading to increased phosphorylation and membrane insertion of GluA1 subunits. Following ectodomain shedding, the C terminal fragment of ICAM-5 could undergo additional processing followed by internalization and degradation. It is worth noting that following MMP or A disintegrin and metalloproteinase (ADAM) mediated shedding, select CAMs are further processed by gamma secretase. Intracellular domains (ICDs) thus generated may be degraded or, in some cases, influence gene transcription [47,48].

## Discussion

Previous studies have shown that MMPs play a role in varied forms of learning and memory (reviewed in 2,3,49–52). Though MMPs cleave varied relevant substrates, including pro-neurotrophins and insulin like growth factor binding proteins [53,54], several studies suggest that their potential to generate integrin-binding ligands likely represents an important means by which they enhance neurotransmission [7,8,21,33]. For example, β_1_ integrin signaling has been implicated in MMP dependent effects on LTP [7,8].

CAMs represent an important class of integrin binding ligands, and their shed ectodomains may interact with previously unengaged integrins to stimulate enhanced NMDAR subunit phosphorylation/function and/or actin polymerization with dendritic spine expansion [28]. In previous work, we have shown that neuronal activity stimulates rapid MMP dependent shedding of the ICAM-5 ectodomain [11], and that recombinant ectodomain can stimulate β_1_ integrin dependent phosphorylation of cofilin [33], an event permissive for dendritic actin polymerization and observed with spine expansion and LTP [21]. We have also observed that soluble ICAM-5 stimulates a β_1_ integrin dependent increase action potential frequency in hippocampal neurons, an endpoint that may be associated with pre and/or post synaptic changes including an increase in the amplitude or frequency of AMPA mEPSCs [32].

In the present study, we find that soluble ICAM-5 stimulates an increase in the frequency, but not the amplitude, of AMPAR mini EPSCs. This is an effect that could follow, at least in part, from post synaptic changes associated with an increase in the number of responsive units. We did not, however, observe an ICAM-5 associated increase in spine number nor in PSD-95 positive puncta (not shown). Another possibility to account for frequency increases would be unsilencing of previously silent, GluA deficient, synapses. It has been suggested that GluA1 containing receptors in particular are inserted into dendritic spines during unsilencing of synapses by electrical stimulation [55], and prior studies have shown that unsilencing of synapses can be associated with increased AMPAR mEPSC frequency [56]. A study focused on cofilin mediated actin dynamics with cLTP also showed an increase in GluA1 insertion into spines that was associated with an increase in mEPSC frequency [57]. Of interest is that a large portion of the spines (50%) that showed increased GluA1 insertion were not measurably enlarged. Post synaptically silent synapses are relatively prevalent in DIV 14 neuronal cultures [58], and also occur in mature brain [59]. As opposed to an increase in mEPSC amplitude, an increase in frequency might be expected if ICAM-5 were to have predominant effects on relatively thin, AMPAR-deficient spines. One possibility is that β_1_ integrins are more highly expressed, or present in a more avid form, on relatively less mature spines. These integrins do localize to synapses in CA1 where they are concentrated postsynaptically [24]. In a recent study, however, while β_1_ integrins were observed on the heads of filopodia, expression was more robust on mature spines [26]. Avidity and ligand binding availability issues as a function of maturity have yet to be fully explored. While integrin dependent effects on actin dynamics in spines from DIV 14 hippocampal neurons from E (15,16) mouse embryos have been demonstrated [25], experiments with acute hippocampal slices prepared at early and later (P21 versus P42) post natal stages suggest that integrin dependent effects on dendritic arbor and synapse stability may be important at relatively later post natal ages [60,61]. ICAM-5 expression as a function of maturity should also be considered. *In vivo*, ICAM-5 expression is higher on thin spines than it is on relatively mature mushroom spines [29]. Thus, *in vivo* shedding might be more likely to present greater agonist levels to relatively thin spines.

The lack of a substantial effect of ICAM-5 on the amplitude of AMPA mEPSCs in the present study is also of interest. While mechanisms including activation of previously silent synapses could contribute to frequency changes, ICAM-5 dependent actin polymerization and spine expansion may not have occurred to an extent sufficient to measurably increase amplitude. Whether ICAM-5 has appreciable effects on the size of small and/or large spines remains to be determined. Of interest is that ICAM-5 did stimulate a non-significant trend towards an increase in filopodia formation, and though filopodia would not be expected to contribute to the increase in mEPSC frequency, this trend is consistent with previous reports showing that select MMPs and integrin binding ligands may influence the morphology of dendritic protrusions [25,62] In particular, a recent study showed that chemical LTP could stimulate rapid, MMP-dependent, development of spine head protrusions and that spines with protrusions gained post synaptic GluAs [62]. It is also of interest that theta burst stimulation has been linked to changes in the morphology of dendritic protrusions with a short lived increase in dendritic filopodia at 30 minutes post-stimulation, and an increase in the width of existing spines that is notable at 2h post-stimulation [63].

It should be mentioned that an increase in mini frequency can also reflect an increase in the probability of neurotransmitter release. We could not perform paired pulse facilitation (PPF) experiments with neuronal cultures and due to the relatively large size of soluble ICAM-5, paired pulse experiments in slices were not pursued. Previous studies that have examined β_1_ integrin signaling in synaptic plasticity have demonstrated defects in AMPAR transmission and LTP [64,65] but no changes in PPF [65]. Of interest, mice with a postnatal knock down of β_1_ had a phenotype similar to animals with knock outs of GluA1 [65]. Thus, while we cannot conclusively rule out the possibility that ICAM-5 might also stimulate a change in transmitter release probability, our subsequent experiments followed up on the potential for ICAM-5 to stimulate changes in the phosphorylation and surface expression of GluA1, a subunit that has been linked to post synaptic effects including activation of post synaptically silent synapses [56,66].

Consistent with a post synaptic locus for mEPSC frequency results, ICAM-5 stimulated an increase in membrane levels of GluA1, and an increase serine 845 phosphorylation of this subunit. Prior studies suggest that serine 845 phosphorylation and synaptic incorporation of GluA1 can occur with LTP [67–70], fear memory [44], and unsilencing of synapses by electrical stimulation [55]. GluA subunit phosphorylation can influence both receptor function and subunit exocytosis [71]. Synaptic incorporation of GluA1 has been shown to increase with LTP as a consequence of subunit movement by lateral diffusion [67]. This is followed, minutes later, by exocytosis of intracellular GluA1 primarily onto the dendritic shaft and potentially to replenish pools for future lateral movement [67]. GluA2 is also important to LTP, and though we did not observe increased membrane levels of GluA2 or examine the phosphorylation or specific function of this subunit, calcium impermeable GluA2 containing receptors could be incorporated into the membrane on a different timescale and/or in response to ICAM-5/integrin independent events that follow LTP induction [72].

While LTP and learning likely increase GluA1 insertion through varied mechanisms including several that are MMP independent, our results suggest that soluble ICAM-5 might also contribute. Though future studies will be necessary to determine mechanisms by which ICAM-5 can influence GluA1 phoshorylation and insertion, integrin signaling has recently been linked to the activation of protein kinase A [45,73], a kinase that can phosphorylate the GluA1 subunit at serine 845 [71]. Non-mutually exclusive mechanisms by which the ICAM-5 ectodomain could contribute to changes in the phosphorylation of GluA1 include integrin dependent phosphorylation of GluN subunits [23] to increase NMDA receptor mediated calcium influx and subsequent GluA1 phosphorylation. Another possibility, though yet untested, is that a kinase more typically linked to integrin signaling, such as Akt, might phosphorylate GluA1.

In summary, we have shown that in DIV 14 neurons, the soluble ICAM-5 ectodomain can increase membrane levels of GluA1 and glutamatergic transmission as determined by a significant increase in the frequency of mEPSCs. If soluble ICAM-5 has the same effects *in vivo*, it could belong to a subset of synaptic CAMs that are shed in a neuronal activity dependent manner to enhance excitatory neurotransmission. Its *in vitro* effects and *in vivo* expression on plasticity spines of the telencephalon make it a molecule of interest that warrants further study. Future studies using CAM cleavage resistant mutants, and unbiased approaches to determine which CAM ectodomains show increased co immunoprecipitation with integrins in the setting of LTP, may be indicated.
